# Purtscher-like retinopathy and paracentral acute middle maculopathy associated with improper antihypertensive drug use: a case report

**DOI:** 10.3389/fmed.2024.1394614

**Published:** 2024-10-18

**Authors:** Yang Meng, Abdulla Sawut, Miao Tian, Ying Li, Liwei Cai, Di Xiao, Zuohuizi Yi, Changzheng Chen

**Affiliations:** ^1^Eye Center, Renmin Hospital of Wuhan University, Wuhan, China; ^2^Physical Examination Center, Renmin Hospital of Wuhan University, Wuhan, China

**Keywords:** Purtscher-like retinopathy, paracentral acute middle maculopathy, hypotension, vision loss, case report, review

## Abstract

**Background:**

Purtscher-like retinopathy (PLR) is a rare retinal microangiopathy with unclear pathogenesis. Paracentral acute middle maculopathy (PAMM) is an optical coherence tomography (OCT) sign proposed in recent years, which is characterized by infarction of the middle layer of the retina. This article reported a rare case of PRL and PAMM probably related to improper antihypertensive drug use in a middle-aged male.

**Case presentation:**

A 49-year-old man presented with a complaint of sudden-onset vision loss and paracentral scotomas in the right eye for approximately 1 week. At presentation, the best-corrected visual acuity (BCVA) was 20/63 OD and 20/20 OS. Fundus examination showed multiple cotton-wool spots and Purtscher flecken in the posterior segment of the right eye. OCT revealed hyper-reflectivities in the inner nuclear layer (INL), consistent with PAMM. En face OCT showed PAMM’s characteristic “fern-like” perivenular changes. Fluorescein angiography demonstrated prolonged arm-to-retina time, delayed artery and venous filling, and hypofluorescence corresponding to cotton-wool spots. Examinations of the left eye were unremarkable. Many imaging and laboratory tests were performed to detect the possible cause of PLR and PAMM, but no possible explanation was found except improper antihypertensive drug use. The patient was recommended to stop his antihypertensive medication, and prescribed other systemic medicines, including oral prednisolone (40 mg q.d. with gradual tapering), oral cobalamin (0.5 mg t.i.d.), and subcutaneous injections of compound anisodine (2.0 mL q.d.) beside the superficial temporal artery. Two weeks after onset, his BCVA improved to 20/25 in the right eye. During follow-ups, his BCVA recovered to 20/20, accompanied by the regression of fundus lesions. The patient reported no treatment-related adverse effects.

**Conclusion:**

This is the first reported case of PLR and PAMM following improper antihypertensive drug use. Our report expands our understanding of the etiology and pathophysiology of PLR and PAMM. We also stress the importance of proper application of medications in clinical practice.

## 1 Introduction

Purtscher’s retinopathy, also known as retinal teletraumatism or traumatic retinal angiopathy, is a rare retinal microangiopathy. It was first described by Austrian ophthalmologist Otmar Purtscher in 1910 (1). He described a man who experienced bilateral vision loss after falling off a tree onto his head. Under the ophthalmoscope, Purtscher noticed a series of fundus abnormalities in the patient, including multiple retinal hemorrhages and retinal whitening (Purtscher flecken) in the posterior poles (1). Consequently, the term “Purtscher retinopathy” emerged to describe similar ocular findings triggered by severe traumas, especially traffic accidents with crush injuries (2–4). Nevertheless, it is noteworthy that some patients may present with characteristic fundus changes of Purtscher retinopathy without a history of preceding traumatic events. This distinction gave rise to the term “Purtscher-like retinopathy (PLR),” which is used to describe similar retinopathies that occur in the absence of traumas (2).

Reported triggers of PLR include systemic lupus erythematosus (SLE), HELLP syndrome (hemolysis, elevated liver enzymes, and low platelet count syndrome), acute pancreatitis, dermatomyositis, COVID-19, breast filler injection, and others (5–10). Patients with Purtscher’s retinopathy and PLR typically experience acute unilateral or bilateral vision loss within 1 to 2 days after the onset of triggering events (2). Common fundus abnormalities comprise cotton-wool spots, Purtscher flecken, and retinal hemorrhages (3). Besides, optic disk swelling and a pseudo-cherry red spot in the macula may also be seen (4). The cotton-wool spots (soft exudates) are due to the localized infarcts of the retinal nerve fiber layer (RNFL). The Purtscher flecken is more characteristic, appearing as multiple, pleomorphic, and clearly demarcated areas of inner retinal whitening between the arteries and veins. Retinal hemorrhages are usually mild and may not exist in some patients. If the fovea is encircled by the whitening retina, a pseudo-cherry red spot becomes visible, mimicking the cherry red spot seen in central retinal artery occlusion (11).

Paracentral acute middle maculopathy (PAMM) is an optical coherence tomography (OCT) sign that is characterized by hyper-reflective changes (i.e., infarction) of the middle layer of the retina (12). PAMM can be caused by local retinal vascular diseases (mostly vascular occlusive disorders), systemic diseases (e.g., sickle cell disease and hypercoagulable conditions), or surgical procedures (ocular and systemic) (12).

As far as we know, there are no reported cases of PLR and PAMM resulting from improper use of antihypertensive medication. In this report, we present a unique case of acute-onset unilateral PLR and PAMM caused by improper administration of antihypertensive drugs in a middle-aged man.

## 2 Case presentation

A 49-year-old man was referred to our hospital with a complaint of sudden-onset vision loss and paracentral scotomas in the right eye, which persisted for approximately 1 week. The patient reported no history of trauma, surgery, ocular diseases, or familial disorders. His only remarkable past medical history was hypertension of approximately 2 years.

**Figure 1:**
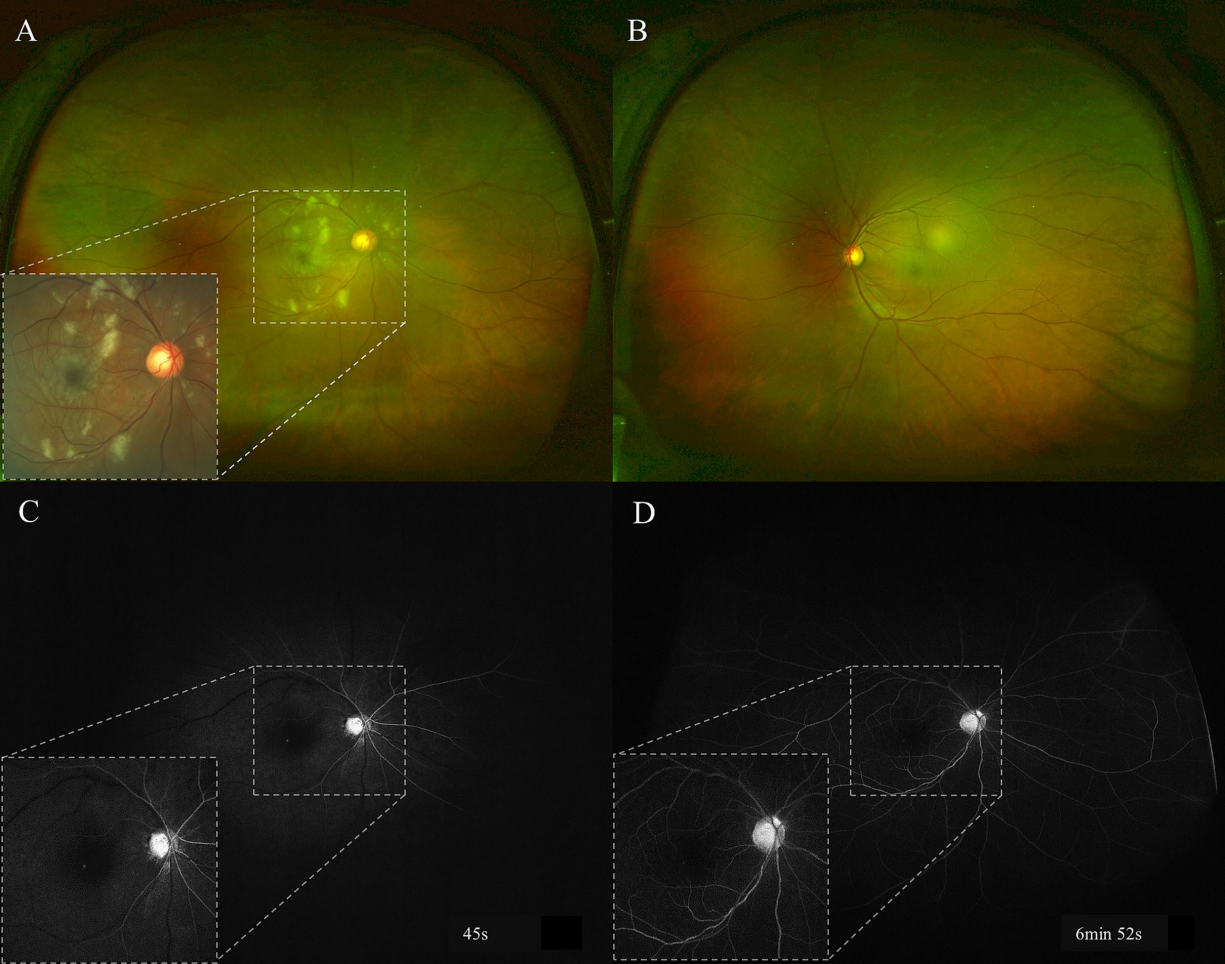
Baseline fundus photography and FFA examinations. There were multiple cotton-wool spots and retinal whitening in the posterior pole of the right eye **(A)**, and these abnormalities can be seen more clearly in color fundus photography (inset). The left fundus was unremarkable **(B)**. The early phase FFA **(C)** showed prolonged arm-to-retina time (around 30s), delayed artery and venous filling, and scattered hypofluorescence due to cotton-wool spots. The hypofluorescence persisted till the late phase **(D)**. FFA: fundus fluorescein angiography.

**Figure 2:**
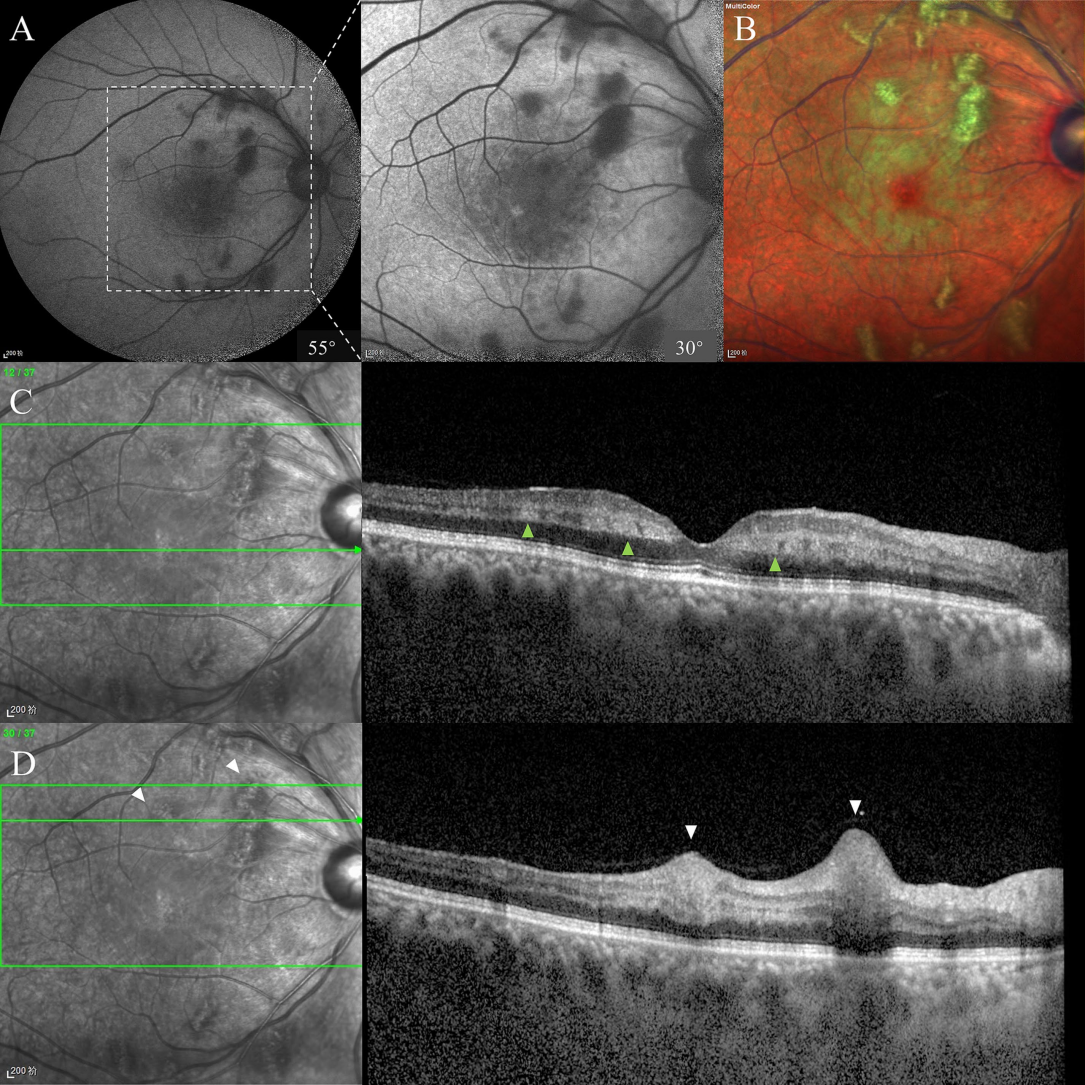
Baseline AF, MultiColor, and OCT examinations. There were macular hypo-AF and blockages of cotton-wool spots on AF, and the details could be better seen in the 30° image **(A)**. A pseudo-cherry red spot could be seen against the whitening macula on MultiColor imaging **(B)**. OCT across the fovea showed INL hyper-reflectivities, consistent with PAMM **(C)**. RNFL infarctions and swellings were also noticed, consistent with cotton-wool spots **(D)**. AF, autofluorescence; OCT, optical coherence tomography; INL, inner nuclear layer; PAMM, paracentral acute middle maculopathy; RNFL, retinal nerve fiber layer.

**Figure 3:**
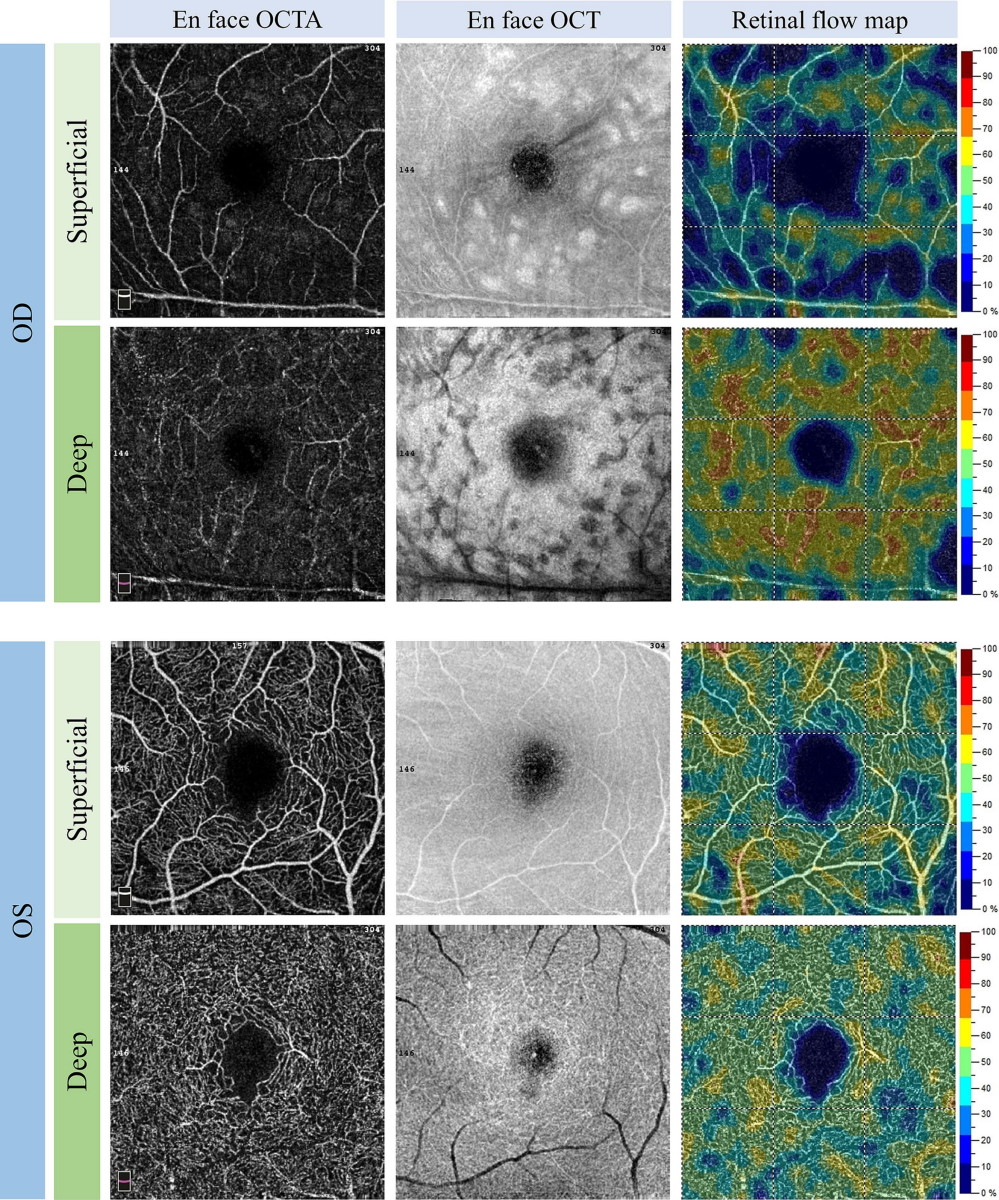
Baseline 3 × 3 mm OCTA examination. OCTA showed reduce in the blood flow of both the superficial and deep labs of the right eye. The “fern-like” perivenular changes were shown on deep lab En face OCT, consistent with PAMM. Note the shadowing caused by the cotton-wool spots in the right eye. OCTA, optical coherence tomography angiography; OCT, optical coherence tomography; PAMM, paracentral acute middle maculopathy.

At presentation, the best-corrected visual acuity (BCVA) was 20/63 in the right eye and 20/20 in the left eye. Intraocular pressure was normal for both eyes. The anterior segments were normal except slight lens opacity in both eyes. Fundus examination revealed several scattered cotton-wool spots, multiple focal retinal whitening in the posterior retina in the right eye (Figure 1A), and a unremarkable finding in the left eye (Figure 1B). Fundus fluorescein angiography (FFA) revealed a prolonged arm-to-retina time, delayed artery and venous filling, and subtle hypofluorescence corresponding to the cotton-wool spots (Figures 1C,D). Autofluorescence (AF) showed macular hypo-AF and blockages of cotton-wool spots near the superotemporal and inferotemporal vascular arcades (Figure 2A). On MultiColor imaging, a pseudo-cherry red spot was seen (Figure 2B). Spectral-domain OCT revealed several isolated hyper-reflective bands involving the middle retina (from outer plexiform layer through inner plexiform layer) of the macula with fovea spared (Figure 2C). Additionally, OCT clearly identified the cotton-wool spots as RNFL swellings (Figure 2D). Further optical coherence tomography angiography (OCTA) examination showed a remarkable reduction in the blood flow of the superficial lab of the right eye in comparison with the left eye (Figure 3). Notably, PAMM’s characteristic “fern-like” perivenular changes were visibly captured in the deep lab of En face OCT (Figure 3).

Based on the patient’s clinical presentations and ophthalmic examinations, a diagnosis of PLR and PAMM was made. Since both PLR and PAMM can be caused by systemic diseases, detailed systemic examinations were performed for the patient. Many imaging and laboratory tests were performed to detect the possible cause of PLR and PAMM, but no possible explanation was found. According to the patient’s condition, oral glucocorticoids (prednisolone; 40 mg q.d. with a gradual tapering schedule) and cobalamin (0.5 mg t.i.d.) were administered. Simultaneously, the patient underwent subcutaneous injections of compound anisodine, beside the superficial temporal artery (2.0 mL q.d.). The patient weighed 75 kg at the time of treatment.

While we were still searching for the cause of our patient’s vision loss, a particular manifestation of his caught our attention. The patient stated that over the past few weeks, he had frequently experienced transient dizziness and bilateral blurred vision. These symptoms could occur multiple times a day, often triggered by suddenly standing up after extended periods of sitting or squatting, and would quickly resolve. He assumed these were common occurrences for someone of his age. However, on this occasion, following a prolonged toilet squat, his vision did not recover as it had previously. We promptly measured his blood pressure, revealing a resting rate of only 98/70 mmHg. Further inquiry revealed that 2 months prior, without consulting a doctor, he had switched his blood pressure medication from benazepril (5 mg q.d.) to a compound formula (amlodipine 5 mg + benazepril 10 mg q.d.). It was after this change that he began experiencing recurrent transient dizziness and vision loss. We recognized that his previous symptoms could be due to postural hypotension (orthostatic hypotension). With close monitoring of the patient’s blood pressure, the patient was recommended to stop his antihypertensive medication. Surprisingly, even after 3 days without the medication, the patient’s resting blood pressure remained within the range of 110–135/75–90 mmHg. Referring to the NICE (National Institute for Health and Care Excellence) guidelines, his blood pressure levels did not seem to meet the criteria for being classified as “high blood pressure” (13). We proceeded with the patient’s treatment using prednisolone, cobalamin, and compound anisodine. One week after his initial presentation (2 weeks after onset), his BCVA improved to 20/25 in the right eye.

**Figure 4:**
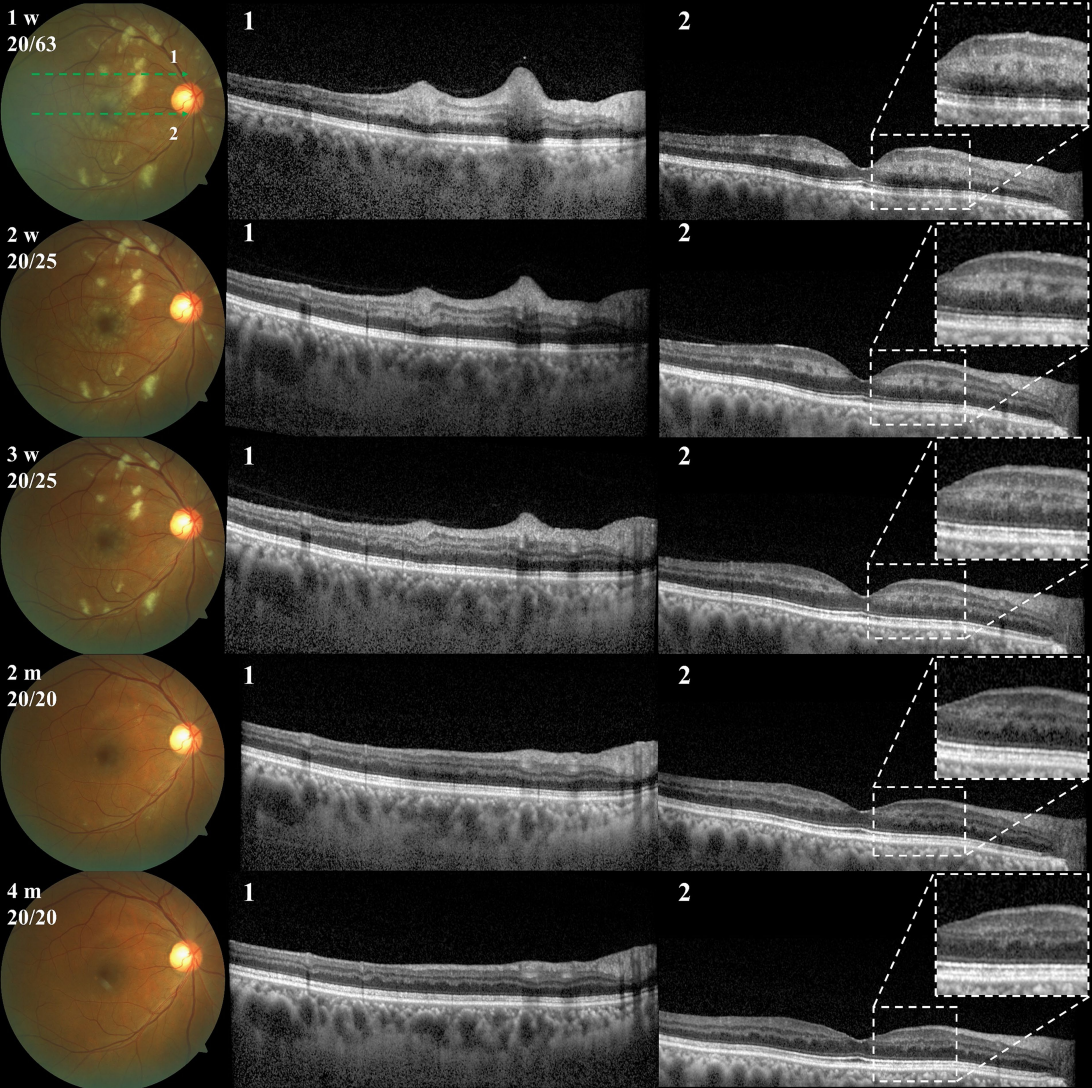
Fundus photography and OCT examinations during follow-up. First row: fundus photographs, with corresponding examination times (from onset) and BCVA in the upper left corner. Second row: OCT scan 1 at the corresponding time points. Third row: OCT scan 2 at the corresponding time points. OCT, optical coherence tomography; BCVA, best-corrected visual acuity.

Three weeks after the patient’s initial vision loss, the BCVA remained stable. Fundus photography revealed partial absorption of the cotton-wool spots and Purtscher flecken (Figure 4). Over this period, several measurements of the patient’s resting blood pressure were within the normal range without any antihypertensive drug use, and his symptoms of postural hypotension did not recur. Thus, the final diagnosis of our patient was PLR and PAMM related to improper antihypertensive drug use. Oral prednisolone was stopped, whereas the use of compound anisodine and cobalamin were stopped at 4 weeks and 3 months after onset, respectively. The patient did not report any treatment-related adverse effects.

During the four-month follow-up period, the patient’s BCVA fully recovered to 20/20. The fundus cotton-wool spots and Purtscher flecken completely resolved, while the PAMM-associated INL hyperreflective structures gave way to INL atrophy (Figure 4). The “fern-like” changes gradually disappeared on En face OCT, accompanied by improved retinal blood perfusion (Supplementary Figure 1). Despite reporting the presence of small paracentral relative scotomas, the patient expressed high satisfaction with the treatment effect. Additionally, we referred the patient to the cardiology department for cardiovascular health status assessment. The patient was diagnosed with “high-normal blood pressure,” according to the 2020 International Society of Hypertension Global Hypertension Practice Guidelines (14). He received recommendations for lifestyle adjustments to naturally lower his blood pressure.

## 3 Discussion

To our knowledge, this is the first reported case of PLR and PAMM possibly related to improper anti-hypertensive drug use. Although the patient did not present until 1 week after onset, the typical features of PLR, i.e., cotton wool spots and Purtscher flecken, could be identified in the posterior pole of the right eye. OCT B scan and En face OCT showed band-like INL hyper-reflectivities and fern-like” perivenular changes, respectively, both of which are characteristic of PAMM.

We postulate that, since the doses of the antihypertensive drug he had previously taken were small (benazepril 5 mg), he did not exhibit symptoms of orthostatic hypotension (i.e., insufficient blood supply to the brain and eyes). However, the patient switched his medication to a more potent combination of antihypertensive medication (amlodipine 5 mg + benazepril 10 mg) without a blood pressure assessment. It was since then that patients began to experience frequent symptoms of blurry vision and dizziness upon standing. Fluorescein angiography showed a prolonged arm-to-retina time as well as delayed artery and venous filling. In addition, orthostatic hypotension did not recur after the patient stopped taking antihypertensive medications, and his blood pressure did not meet the criteria for hypertension. Therefore, it is very likely that his PLR and PAMM were caused by insufficient blood supply to the eye due to inappropriate use of antihypertensive drugs.

The pathogenesis of Purtscher and Purtscher-like retinopathies has not been fully understood. In the initial report, Purtscher hypothesized that the retinal changes were due to a sudden increase in intracranial pressure resulting in lymphatic extravasation of retinal blood vessels (2). Since then, a wide variety of mechanisms have been proposed. For instance, in patients suffering from traumatic injuries, Purtscher retinopathy is believed to be caused by microembolization of fat released from damaged tissue into the systemic circulation, as well as vasculitis triggered by free fatty acids and lipase (15–17). In cases who developed Purtscher flecken due to asphyxia, weight-lifting, chest compression, and battered baby syndrome (also known as shaken baby syndrome), impaired venous return and acute expansion of retinal veins are thought to be responsible for the retinal changes (2, 18). In pancreatitis-induced PLR, the release of activated proteases is thought to be the culprit (19). Besides, it has been suggested that in SLE, complement activation, lymphocyte adhesion, and vasculitis-related endothelial damage could result in retinal arteriolar microembolization, thereby causing PLR (20).

Drug use as a potential trigger for PLR has attracted increasing attention. Reported possible triggers of PLR include dupilumab treatment for atopic dermatitis, gemcitabine therapy for lung cancer, drug-induced hypersensitivity syndrome, antineoplastic therapy for Hodgkin’s lymphoma, hyaluronic filler injection for facial and breast augmentation, and peribulbar/retrobulbar anesthesia before ocular surgeries (10, 21–27). However, it should be noted that since PLR is a rare disease, the aforementioned triggers were mainly reported in sporadic case studies, and their role in inducing PLR still requires further investigation.

Here, our case provided a new idea for the mechanism of PLR. Specifically, low blood pressure caused insufficient brain and ocular blood supply in our patient. The former was responsible for his dizziness, whereas the latter caused the multi-level retinal ischemia and infarctions, manifesting as cotton-wool spots and PAMM. Cotton-wool spots are ischemic signs on the superficial retina, which are believed to be associated with many systemic diseases that affect blood vessels, such as diabetes and hypertension (28). PAMM is also a manifestation of retinal ischemia in the deep vascular complex (i.e., the intermediate capillary plexus and deep capillary plexus) (12, 29). The simultaneous occurrence of cotton-wool spots, PAMM, and PLR in this patient also indicates, at least to some extent, that his ocular manifestations were caused by ocular ischemia (hypoperfusion).

An interesting finding is that although Purtscher’s retinopathy and PLR usually affect both eyes, unilateral involvement is not uncommon (30–32). One possible explanation is that there may be potential asymmetry in Purtscher’s retinopathy and PLR (33). This could explain why some patients only have unilateral involvement and why the severity of the disease may vary between both eyes even in patients with bilateral involvement. However, this theory needs further investigation.

In summary, we have presented a rare case of PLR and PAMM following improper antihypertensive drug use. The patient recovered well after treatment. This report broadens our comprehension of the etiology and pathophysiology underlying PLR and PAMM. Furthermore, this case underscores the importance of proper application of medications in clinical practice.

## Data Availability

The original contributions presented in the study are included in the article/Supplementary material, further inquiries can be directed to the corresponding authors.

## References

[1] Kehler A. Optic atrophy and retinal changes following thoracic trauma; survey of the relationship between traumatic retinal angiopathy (Purtscher), traumatic asphyxia and fat embolism and report of a case. Acta Ophthalmol. (1953) 31:437–56. doi: 10.1111/j.1755-3768.1953.tb07662.x, PMID: 13138079

[2] Agrawal A McKibbin MA. Purtscher's and Purtscher-like retinopathies: a review. Surv Ophthalmol. (2006) 51:129–36. doi: 10.1016/j.survophthal.2005.12.003 16500213

[3] Agrawal A McKibbin M. Purtscher's retinopathy: epidemiology, clinical features and outcome. Br J Ophthalmol. (2007) 91:1456–9. doi: 10.1136/bjo.2007.117408, PMID: 17556428 PMC2095457

[4] Miguel AI Henriques F Azevedo LF Loureiro AJ Maberley DA. Systematic review of Purtscher's and Purtscher-like retinopathies. Eye (Lond). (2013) 27:1–13. doi: 10.1038/eye.2012.222, PMID: 23174749 PMC3545384

[5] Meng L Yu Q Zhao X Chen L Wang Y Zhang W. Purtscher-like retinopathy in systemic lupus erythematosus: clinical features, risk factors and prognosis. QJM. (2023) 116:923–32. doi: 10.1093/qjmed/hcad204, PMID: 37665730

[6] Tsui JC Kolomeyer AM. HELLP syndrome-associated Purtscher-like retinopathy. Ophthalmology. (2023) 130:1161. doi: 10.1016/j.ophtha.2022.11.011 36517280

[7] Narang S Aggarwal P Bhattacharyya A Singla S Negi R. Acute pancreatitis presenting as Purtscher-like retinopathy. Pancreatology. (2022) 22:333–4. doi: 10.1016/j.pan.2022.01.005, PMID: 35090821

[8] Yan Y Shen X. Purtscher-like retinopathy associated with dermatomyositis. BMC Ophthalmol. (2013) 13:36. doi: 10.1186/1471-2415-13-36, PMID: 23883070 PMC3724690

[9] Pastore MR Furlanis G Tognetto D. Bilateral Purtscher-like retinopathy associated with SARS-CoV-2 infection. JAMA Ophthalmol. (2022) 140:e214979. doi: 10.1001/jamaophthalmol.2021.4979, PMID: 35175295

[10] Pee XK Low A Ab Kahar M Mohamed SO Chong YJ. Purtscher-like retinopathy and paracentral acute middle maculopathy following breast filler injection. BMC Ophthalmol. (2023) 23:444. doi: 10.1186/s12886-023-03186-8, PMID: 37932684 PMC10629129

[11] Fan W Huang Y Zhao Y Yuan R. Central retinal artery occlusion without cherry-red spots. BMC Ophthalmol. (2023) 23:434. doi: 10.1186/s12886-023-03176-w, PMID: 37880636 PMC10601202

[12] Scharf J Freund KB Sadda S Sarraf D. Paracentral acute middle maculopathy and the organization of the retinal capillary plexuses. Prog Retin Eye Res. (2021) 81:100884. doi: 10.1016/j.preteyeres.2020.100884, PMID: 32783959

[13] Boffa RJ Constanti M Floyd CN Wierzbicki AS Guideline C. Hypertension in adults: summary of updated NICE guidance. BMJ. (2019) 367:l5310. doi: 10.1136/bmj.l5310, PMID: 31636059

[14] Unger T Borghi C Charchar F Khan NA Poulter NR Prabhakaran D. 2020 International Society of Hypertension Global Hypertension Practice Guidelines. Hypertension. (2020) 75:1334–57. doi: 10.1161/HYPERTENSIONAHA.120.15026 32370572

[15] Roden D Fitzpatrick G O'Donoghue H Phelan D. Purtscher's retinopathy and fat embolism. Br J Ophthalmol. (1989) 73:677–9. doi: 10.1136/bjo.73.8.677, PMID: 2765450 PMC1041845

[16] Chuang EL Miller FS 3rd Kalina RE. Retinal lesions following long bone fractures. Ophthalmology. (1985) 92:370–4. doi: 10.1016/s0161-6420(85)34023-x, PMID: 3991127

[17] Scotton WJ Kohler K Babar J Russell-Hermanns D Chilvers ER. Fat embolism syndrome with Purtscher's retinopathy. Am J Respir Crit Care Med. (2013) 187:106. doi: 10.1164/rccm.201205-0881IM, PMID: 23281351

[18] Kocak N Kaynak S Kaynak T Oner HF Cingil G. Unilateral Purtscher-like retinopathy after weight-lifting. Eur J Ophthalmol. (2003) 13:395–7. doi: 10.1177/112067210301300412, PMID: 12872799

[19] Tabandeh H Rosenfeld PJ Alexandrakis G Kronish JP Chaudhry NA. Purtscher-like retinopathy associated with pancreatic adenocarcinoma. Am J Ophthalmol. (1999) 128:650–2. doi: 10.1016/s0002-9394(99)00204-4, PMID: 10577543

[20] Wu C Dai R Dong F Wang Q. Purtscher-like retinopathy in systemic lupus erythematosus. Am J Ophthalmol. (2014) 158:e1:1335–1341.e1. doi: 10.1016/j.ajo.2014.09.001, PMID: 25205559

[21] Cheng X Wang F Li H. Purtscher-like retinopathy in a patient of atopic dermatitis associated with Dupilumab use: a case report. Ocul Immunol Inflamm. (2024) 32:779–83. doi: 10.1080/09273948.2023.2192277, PMID: 37442378

[22] Banach MJ Williams GA. Purtscher retinopathy and necrotizing vasculitis with gemcitabine therapy. Arch Ophthalmol. (2000) 118:726–7. doi: 10.1001/archopht.118.5.726, PMID: 10815173

[23] Coban-Karatas M Turunc T Altan-Yaycioglu R. Purtscher-like retinopathy related to drug-induced hypersensitivity syndrome. Ocul Immunol Inflamm. (2012) 20:475–7. doi: 10.3109/09273948.2012.714441, PMID: 23163426

[24] Parc C. Purtscher-like retinopathy as an initial presentation of a thrombotic microangiopathy associated with antineoplastic therapy. Am J Hematol. (2007) 82:486–8. doi: 10.1002/ajh.20845, PMID: 17211841

[25] Serkies-Minuth E Glasner P Michalska-Malecka K Baranska-Rybak W. Purtscher-like retinopathy after hyaluronic filler injection for facial augmentation. J Eur Acad Dermatol Venereol. (2024) 38:e79–81. doi: 10.1111/jdv.19439, PMID: 37595784

[26] Narendran S Saravanan VR Pereira M. Purtscher-like retinopathy: a rare complication of peribulbar anesthesia. Indian J Ophthalmol. (2016) 64:464–6. doi: 10.4103/0301-4738.187679, PMID: 27488158 PMC4991182

[27] Blodi BA Williams CA. Purtscher-like retinopathy after uncomplicated administration of retrobulbar anesthesia. Am J Ophthalmol. (1997) 124:702–3. doi: 10.1016/s0002-9394(14)70917-1, PMID: 9372733

[28] Schmidt D. The mystery of cotton-wool spots - a review of recent and historical descriptions. Eur J Med Res. (2008) 13:231–66. 18558551

[29] Moura-Coelho N Gaspar T Ferreira JT Dutra-Medeiros M Cunha JP. Paracentral acute middle maculopathy-review of the literature. Graefes Arch Clin Exp Ophthalmol. (2020) 258:2583–96. doi: 10.1007/s00417-020-04826-1, PMID: 32661700

[30] Ganne P Slesser D Syamala DD. Purtscher-like retinopathy in systemic lupus erythematosus. QJM. (2023) 116:233–4. doi: 10.1093/qjmed/hcac249, PMID: 36321999

[31] Shroff D Kumar S Naidu A Gupta C. Unilateral Purtscher-like retinopathy post-COVID-19. Indian J Ophthalmol. (2022) 70:3710–2. doi: 10.4103/ijo.IJO_1486_22, PMID: 36190079 PMC9789798

[32] Ipek SC Yaman A Men S Saatci AO. Unilateral Purtscher-like retinopathy as the presenting feature of a case with spontaneous carotid artery dissection. Clin Case Reports. (2021) 9:e03633. doi: 10.1002/ccr3.3633, PMID: 34026118 PMC8127545

[33] Ang L Chang BCM. Purtscher-like retinopathy - a rare complication of acute myocardial infarction and a review of the literature. Saudi J Ophthalmol. (2017) 31:250–6. doi: 10.1016/j.sjopt.2017.05.009, PMID: 29234228 PMC5717495

