# Supplementary material for: Purtscher-like retinopathy and paracentral acute middle maculopathy associated with improper antihypertensive drug use: a case report
Source: Front Med (Lausanne). 2024 Oct 18;11:1394614. doi: 10.3389/fmed.2024.1394614 (PMC11527711; doi:10.3389/fmed.2024.1394614)
Supplement: Supplementary file 1 [file Data_Sheet_1.PDF]

Supplementary Material

Supplementary Figure 1

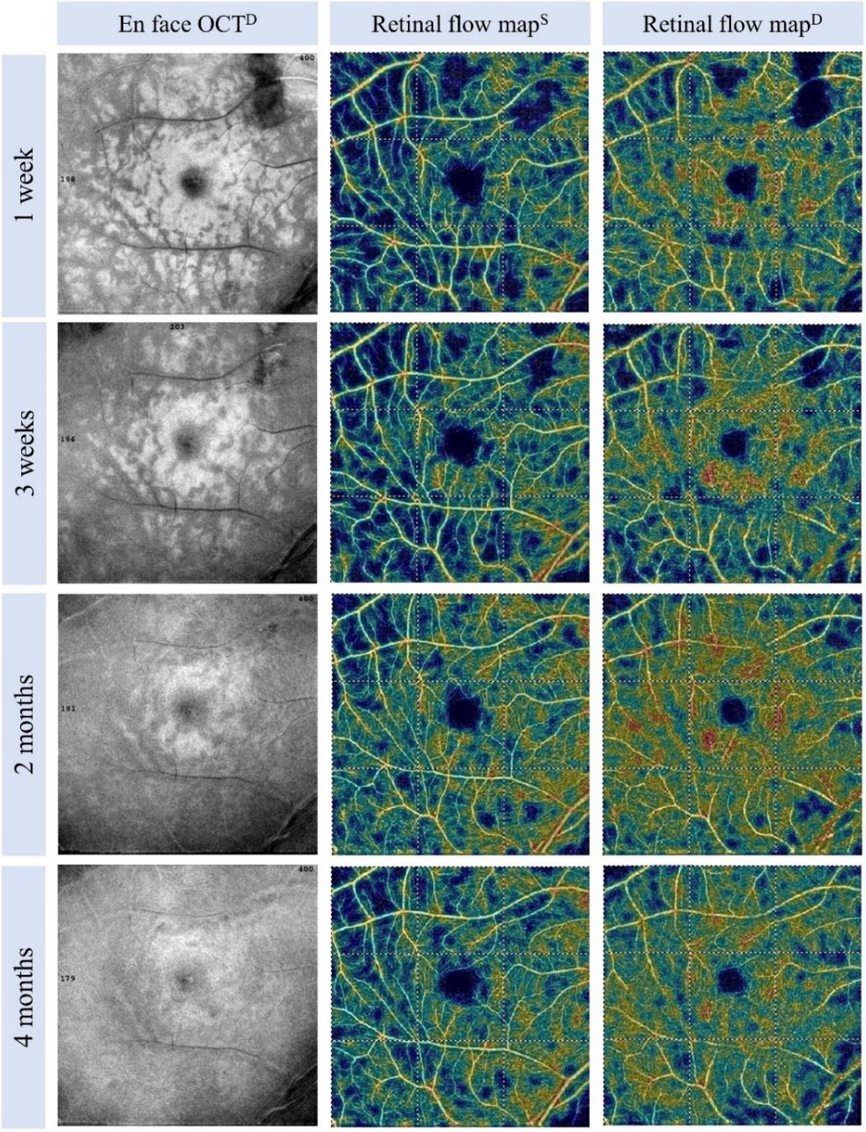

Supplementary Figure 1. OCTA examinations during follow-up.

En face OCT^D: the deep slab of En face OCT. Retinal flow map^S: the superficial slab of retinal flow map. Retinal flow map^D: the deep slab of retinal flow map. OCTA: optical coherence tomography angiography; OCT: optical coherence tomography.
